# Videoconferencing in Pressure Injury: Randomized Controlled Telemedicine Trial in Patients With Spinal Cord Injury

**DOI:** 10.2196/27692

**Published:** 2022-04-19

**Authors:** Ingebjørg Irgens, Jana Midelfart-Hoff, Rolf Jelnes, Marcalee Alexander, Johan Kvalvik Stanghelle, Magne Thoresen, Tiina Rekand

**Affiliations:** 1 Department of Research Sunnaas Rehabilitation Hospital Nesoddtangen Norway; 2 Institute of Clinical Medicine University of Oslo Oslo Norway; 3 Spinal Cord Unit Department of Neurology Haukeland University Hospital Bergen Norway; 4 Faculty of Health VID Specialized University Bergen Norway; 5 Medical Center Hospital Sønderjylland Aabenraa Denmark; 6 Department of Physical Medicine and Rehabilitation Birmingham School of Medicine University of Alabama Birmingham, AL United States; 7 Department of Physical Medicine and Rehabilitation Spaulding Rehabilitation Hospital Harvard School of Medicine Boston, MA United States; 8 Sustain Our Abilities Birmingham, AL United States; 9 Department of Biostatistics University of Oslo Oslo Norway; 10 Sahlgrenska Academy and Institute for Neuroscience and Physiology University of Gothenburg Gothenburg Sweden

**Keywords:** telemedicine, telecommunication, videoconference, outpatient follow-up, spinal cord injury, pressure injury, healing, participant satisfaction, participant interaction, health-related quality of life

## Abstract

**Background:**

Geographical, financial and travel-related barriers may impact access to necessary health care for people in need of long-term follow-up.

**Objective:**

The goal of the research was to perform a nonblinded, randomized, controlled trial on health-related quality of life (HRQoL), healing, interaction, and satisfaction of patients with spinal cord injury (SCI) and PI receiving multidisciplinary videoconference consultations from a wound clinic to the participant’s home versus regular outpatient care. The multidisciplinary team consisted of a medical doctor, a wound nurse, and an occupational therapist. In both groups, district nurses attended the consultations at the participant’s home.

**Methods:**

A total of 56 participants, 28 in each group, were randomized to a videoconference group (VCG) or a regular care group (RCG). Validated questionnaires were used to measure and compare the follow-up effect on HRQoL. Percentage reduction of wound volume was measured at end of the follow-up. A Likert scale was used to measure the satisfaction of the patients and district nurses regarding the interaction between different modalities of care in the 2 groups.

**Results:**

The HRQoL did not show significant differences between the 2 groups (P values ranging from .09 to .88) or the rate of PI healing, experienced interaction, and satisfaction in the groups. A total of 67% (37/55) of all PIs healed, 64% (18/28) in the VCG and 70% (19/27) in the RCG. Mean reduction in ulcer volume was 79% in the VCG and 85% in the RCG (P=.32). A Kaplan-Meier plot with a logrank test regarding time to healing did not show any significant difference between the 2 groups.

**Conclusions:**

Videoconference-based care seems to be a safe and efficient way to manage PIs in terms of HRQoL, healing, interaction, and satisfaction compared to conventional care for people with SCI. This should be considered when planning for future care. SCI has a huge impact on the individual, the family, and the health care system. There is an urgent need to improve systems of care so that individuals who live far from specialists and require long-term follow-up for conditions such as PI can get optimal treatment.

**Trial Registration:**

ClinicalTrials.gov NCT02800915; https://clinicaltrials.gov/ct2/show/NCT02800915 and Current Research Information System in Norway (CRISTIN) 545284; https://app.cristin.no/projects/show.jsf?id=545284

## Introduction

### Background

For people living in rural or medically underserved areas, treatment access may be limited or even nonexistent [1]. Financial situation, travel and treatment costs, ability to take paid time off from work to visit a clinic or hospital, health insurance issues, pandemics, climate change, and unpredictable weather conditions may all impact access to necessary health care [1,2]. Transportation to hospitals and outpatient clinics may be a barrier because of length and duration of the transportation, discomfort, stress, and risks related to the transport [3]. People with spinal cord injury (SCI) are at particular risk of developing pressure injury (PI) due to paralysis, reduced skin sensitivity, and skin exposure to moisture for extended periods of time [4]. They are often hospitalized for long periods of time and need frequent outpatient care for treatment and to monitor the treatment [5]. However, long transport can cause new wounds to develop [6]. This may cause people not to attend to needed appointments [7]. Telecommunication could help to overcome such limitations [2,8]. Telecommunication between hospital and home is a potential way to offer effective health services, regardless of the geographical location of the patient and health care professional [9,10]. Telecommunication in health care covers a broad range of digital remote care services, all with the aim to provide investigation, monitoring, and management of patients and education for patients and staff using technology, allowing access to expert advice and patient information, no matter where the patient or relevant information is located [11]. Different solutions are in use, depending on the health service offered, technology needed, and performance of the service. There are real-time services like videoconferencing, videophone solutions and phone calls, store-and-forward services like text messages and electronic data collection and transmission, and web-based interactive platforms [7,12]. Services can be used to deliver education, consultation, therapy, social support, data collection and monitoring, and clinical care delivery [7,12]. Real-time video consultations allow health care professionals to perform remote visits to the patients’ homes with the possibility to communicate and interact directly with each other [10,11]. Moreover, local care providers, like district nurses, can be included in the consultation. Thus, this system of care delivery increases the possibility of interaction between members at different health care levels and the patient.

### Prior Work

Today there are telecommunication services available for many different health care issues. Teleradiology, telepathology, teledermatology, and telepsychiatry are popular and established areas all with the purpose of transmitting images, test results and medical information, as well as performing evaluations and consultations. The transmitting is via digitalized solutions, video and telephony [7,12-18]. Some services, like cardiology, electrocardiography, ultrasonography and mammography, are available at several hospitals and in different countries, while some services, like emergency medicine, immunology, hematology and speech therapy, are only performed in individual countries or individuals hospitals [7,16,18,19]. As in rehabilitation, research into long-term follow-up has shown mixed evidence of feasibility and efficacy regarding use of telecommunication solutions [15-18,20,21].

The Sunnaas model of telerehabilitation [22] has been used to provide videoconferencing as part of inpatient and outpatient rehabilitation services at a Norwegian rehabilitation hospital. A feasibility study evaluated videoconference as a possible alternative method for outpatient follow-up for patients with SCI and PI [4].

### Goal of the Study

The primary aim of this study was to investigate if videoconference consultations could increase health-related quality of life (HRQoL) in people with SCI and PI. Secondarily, we wanted to determine whether PI healing, perceived interaction, and satisfaction could be considered as good and efficient as conventional follow-up [11].

## Methods

### Recruitment

**Figure 1:**
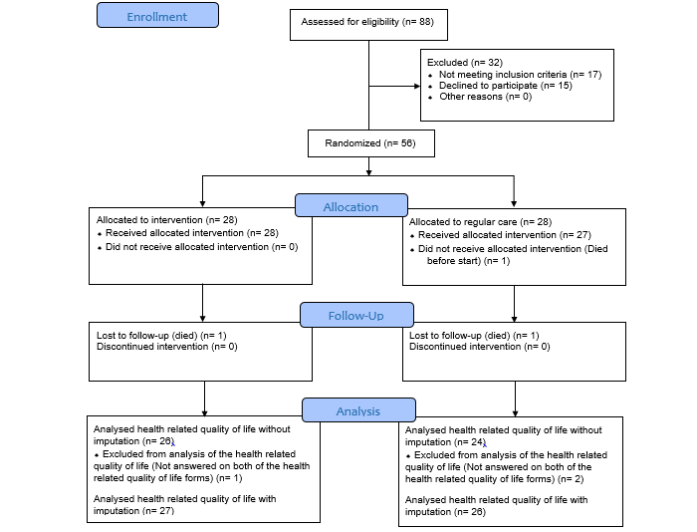
CONSORT 2010 (Consolidated Standards of Reporting Trials 2010) flow diagram of the trial.

People with SCI and ongoing PI were invited to participate in a nonblinded, national, randomized controlled study at 2 spinal cord units in Norway, located at Haukeland University Hospital in western Norway and Sunnaas Rehabilitation Hospital in southeastern Norway. Participants were invited based on response to a questionnaire [11] and from referrals to the outpatient wound clinic at the units. Inclusion criteria were traumatic or nontraumatic SCI, ongoing PI, aged over 18 years, and consent to participate. Individuals were included regardless of concomitant medical concerns. Exclusion criteria were not living in Norway and unable to give their consent due to cognitive impairments. Eligible participants were provided with written and oral information and signed a written consent before inclusion. The study took place between March 6, 2016, and October 19, 2019. The study flowchart is shown in Figure 1.

### Study Design

**Figure 2:**
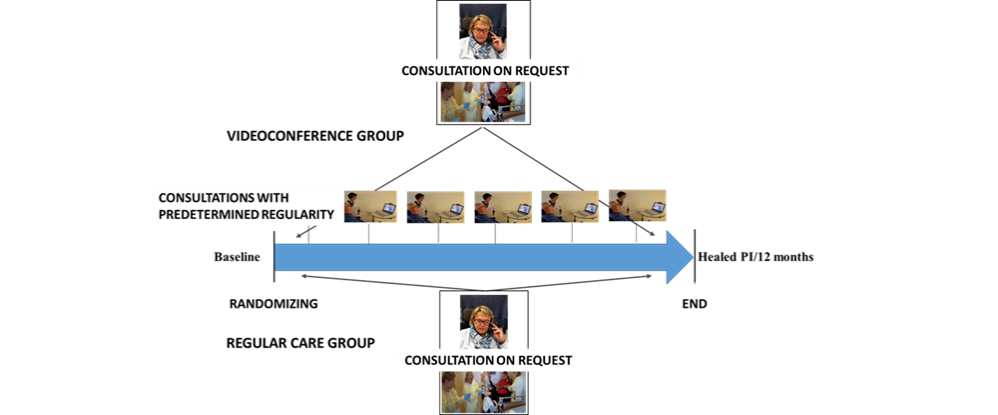
Organization of follow-up for the videoconference and regular care groups.

Once the written consent was given, baseline data were collected and participants were randomized to a videoconference group (VCG) and a regular care group (RCG) by use of the random number generator in the statistical software SPSS (version 25, IBM Corp). The group allocations were then told to the participants. For both groups, a multidisciplinary wound team conducted the follow-up from the outpatient clinic. The team consisted of a medical doctor with several years of experience in PI treatment, a certified wound care nurse, and an occupational therapist with specialized skills regarding pressure measurements and PI follow-up. For both groups, district nurses were present with the participant at the participant’s home during the consultations. The district nurses performed the wound treatment supported by remote guidance from the multidisciplinary wound team at the outpatient clinic. The participants in the RCG received treatment and guidance based on existing routines (ie, by telephone or outpatient consultations at the hospital, if requested). The participants in the VCG were offered treatment and guidance via predetermined videoconference consultations and regular care similar to the RCG. Both groups were followed until healing of the PI or for a maximum of 52 weeks. Figure 2 shows the organization of the follow-up in the 2 groups.

The timeline for study enrollment, intervention, and assessment is described in the Standard Protocol Items: Recommendations for Interventional Trials (SPIRIT) [23]. The Template for Intervention Description and Replication (TIDieR) [24] checklist and guide were used to record and describe the intervention. The study conforms to the Consolidated Standards of Reporting Trials (CONSORT) guidelines extension for randomized pilot and feasibility trials [25].

### Technical Logistics

**Figure 3:**
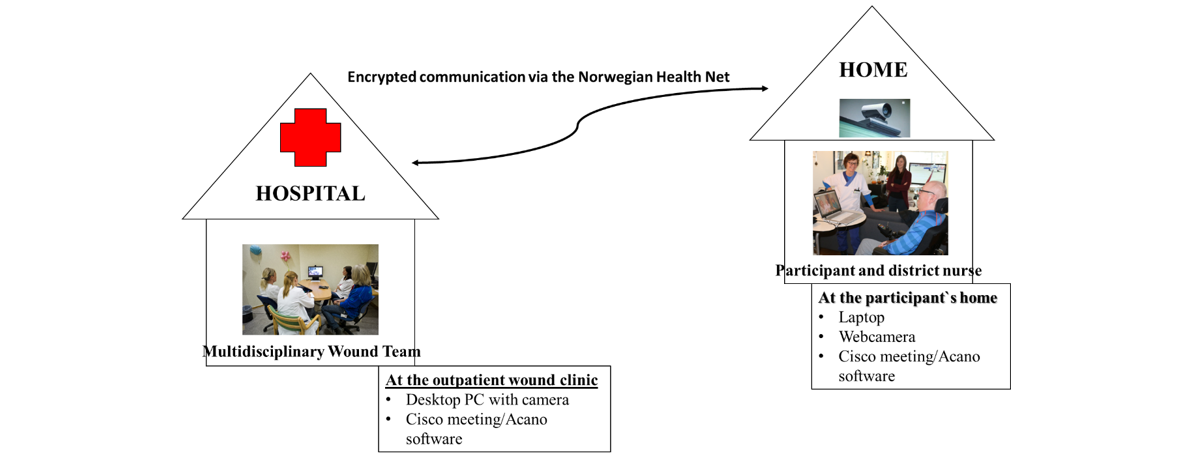
Organization of the videoconference consultations.

In both groups, the district nurses used their work phone or the participant’s cell phones for telephone consultations. For participants in the VCG, arrangements for installation of encrypted software and rehearsal in the use of the program and equipment were addressed immediately after randomization. All participants in the VCG had available broadband or mobile broadband connection. Most of them used their private laptops in the consultations or they borrowed laptops from the hospital’s storage. All of them borrowed mobile webcams from the hospital’s storage. The consultations were performed as synchronous live, videoconferencing in real time, using a Cisco TelePresence System EX90 PC with camera at the wound clinic and a laptop with a mobile webcam at the participant’s location. Encrypted communication channels via the Norwegian Health Net were used to protect privacy of the participants [26]. The wound care nurse at the outpatient wound clinic tested the equipment with the participant and the district nurses before start of the follow-up. Each participant was given a unique subscription number. The wound care nurse at the outpatient wound clinic addressed the participant at each session, and the participant had to accept the call before the consultation could start. Figure 3 shows the organization of the videoconference consultations.

### Information and Guidance

For both groups, the participants gave their consent to send medical records to the general practitioner and their district nurses after each consultation, no matter the kind of consultation. For both groups, all treatment and guiding were conducted in accordance with evidence-based wound therapy guidelines [27] and individualized in accordance with each participant’s needs. The district nurses in both groups were guided in treatment principles according to their knowledge needs. Clinical guidelines, online education programs, and e-learning programs were accessible for the district nurses in both groups.

### Study Variables

Demographic information included gender, date of birth, age at SCI, time since SCI, etiology (traumatic, nontraumatic), level and grade of the SCI, and SCI associated problems. SCI was described according to the International Standards for Neurological Classification of SCI recommendations including clinical findings standardized by the American Spinal Injury Association (ASIA) Impairment Scale (AIS) [28]. Any use of alcohol or tobacco or abuse of drugs was recorded. In addition, information regarding any previous PIs and PI recurrence was recorded, together with the number of present PIs, as well as the category and volume of the present PIs. All PIs were categorized and numbered according to the joint 2019 guideline prepared by the 3 collaborating PI organizations: National Pressure Injury Advisory Panel, European Pressure Ulcer Advisory Panel, and Pan Pacific Pressure Injury Alliance [27]. According to this guideline, the categorization of PIs varies with size and severity of the tissue affected, ranging from reddening of the skin (category 1) to damage to muscle and underlying bone (category 4). In category 1 and 2, the injury is partially going through the skin, while in category 3 and 4, there is a full thickness skin wound. In a suspected deep tissue injury, the depth and severity of the wound is unknown. In an unstageable PI, the wound cannot be categorized due to sloughing/scarring [27]. The PI categorization and volume, (length × width × depth) was measured at baseline by the medical doctor and wound care nurse and at the end of the follow-up by either the medical doctor and wound care nurse at the outpatient wound clinic or by the district nurses guided by the wound team. A ruler adapted for PI measurement was used. The district nurses gained access to the rulers via the multidisciplinary wound team. Difference in volume was calculated as percentage change. Time to healing was measured as days from baseline to healing. Changes in HRQoL in the 2 groups were compared using the Norwegian versions of the 36-item Short Form Health Survey (SF-36) [29] and the Five-Dimension European Quality of Life (EQ-5D) scale [30]. In case of lack of an available Norwegian index version of the EQ-5D scale, the validated UK index is recommended to be used in analyses regarding Norwegian subpopulations [30]. We also used the International Spinal Cord Injury Quality of Life Basic Data Set (ISCI-QoL-BDS) questionnaire [31] to measure the HRQoL among the participants. The form used is similar to the version used by the Norwegian Spinal Cord Injury Registry [32].

The participants reported subjective ratings regarding satisfaction, safety, and level of interaction during the follow-up using a Likert scale with 1 being completely dissatisfied and 5 being totally satisfied. Moreover, as an ad hoc analysis, we wanted to gain knowledge about the district nurses’ experience, and thus we invited them to report their ratings as well.

### Ethics

The research project was carried out in accordance with ethical guidelines and privacy rights for health services in Norway [26] based on the code of ethics of the World Medical Association (Declaration of Helsinki) for experiments involving humans. Established routines to secure confidentiality and ethical guidelines for conducting consultations involving examinations related to intimate body areas, which may be visible on the screen, were established before the study was initiated [22]. Knowledge and expertise achieved through a previous feasibility study [4] was applied in this study. Communication occurred through the Norwegian Health Network’s encrypted video channels. The study was performed in compliance with Norwegian data security and privacy standards [26]. The study was approved by the regional committees for medical and health research (2014/684/REK-Nord) [33] and registered with ClinicalTrials.gov (NCT02800915). All participants were insured through the Norwegian health care system and the hospitals’ insurance programs for adverse effects.

### Statistical Analyses

Demographic variables were descriptively analyzed. Continuous variables are presented as mean with standard deviation whereas categorical variables are presented as counts and percentages. For the HRQoL scores, missing data were handled by multiple imputation. Each missing data point was replaced by m=20 imputed values based on the predictive mean matching technique before analysis. The imputation models include age, gender, AIS grade, and HRQoL scores.

Mean HRQoL scores with corresponding 95% confidence interval are presented for each of the 2 treatment groups at baseline and end of follow-up, and the groups were compared using linear regression analysis with adjustment for baseline. This analysis was repeated without imputation for missing values as well, for comparison. The mean percentage reduction in PI size was calculated with corresponding 95% confidence interval for each of the 2 groups and compared using a Mann-Whitney test. Time to healing was analyzed by the logrank test and is presented by a Kaplan-Meier plot.

P<.05 is considered significant. Independent *t* tests were used to analyze the mean difference in participant satisfaction scores. Corresponding 95% confidence intervals were calculated. All statistical analyses were performed using SPSS statistical software.

### Sample Size

We based our sample size calculation on investigation of HRQoL and the group comparison at the end of follow-up. Our hypothesis was that HRQoL would increase in the VCG as compared to the RCG, and the sample size calculation was based on an expectation of a standardized difference of at least 0.8 (typically considered a large effect). With 80% power, we would need 25 patients in each of the 2 groups.

## Results

### Demographics

A total of 56 participants were included, with 28 in each group. One participant in the RCG died of acute illness prior to start of the follow-up, and the participant’s data were excluded from the analyses. Furthermore, 2 participants, 1 in each group, did not complete any of the HRQoL questionnaires and were removed from the analysis of the primary outcome. Two participants, 1 in each group, died during the follow-up. They are included in the analysis of wound healing as not healed PIs. All deceased participants were male and causes of death were reported to be cardiovascular disease (2) and pneumonia (1). Of the 55 participants included in the analysis, the majority were male, 86% (24/28) in the VCG and 78% (21/27) in the RCG. The mean age was 58 years in both groups. Baseline data of the included participants are shown in Table 1.

**Table 1:** Baseline data of the participants in the 2 groups.

Characteristics		Videoconference group (n=28)		Regular care group (n=27)
**Gender, n (%)**				
	Male	24 (86)	21 (78)	
	Female	4 (14)	6 (22)	
Age (years), mean (SD)		57.50 (14.2)		57.96 (12.81)
**Age group (years), n (%)**				
	15-29	0 (0)	1 (4)	
	30-44	6 (21)	3 (11)	
	45-59	8 (29)	12 (44)	
	60-74	12 (43)	9 (33)	
	75+	2 (7)	2 (7)	
Years since SCI^a^, mean (SD)		16.30 (12.7)		18.90 (15.0)
**Etiology of injury, n (%)**				
	TSCI^b^	22 (79)	24 (89)	
	NTSCI^c^	6 (21)	3 (11)	
**Level of injury^d^, n (%)**				
	C1-C4	4 (14)	5 (19)	
	C5-C8	5 (18)	6 (22)	
	T1-S3	19 (68)	16 (59)	
**AIS grade^e^, n (%)**				
	A	18 (64)	18 (67)	
	B	3 (11)	0 (0)	
	C	6 (21)	8 (30)	
	D	1 (4)	1 (4)	
**SCI-associated problems, n (%)**				
	Incontinence	25 (89)	23 (85)	
	Pain (all types)	8 (29)	9 (33)	
	Spasticity	9 (32)	8 (30)	
PI^f^ category, mean (SD)		2.90 (0.86)		2.82 (0.98)
**Other PIs/PI recurrence, n (%)**				
	No	3 (11)	7 (26)	
	Yes, other PI	11 (39)	9 (33)	
	Yes, recurrence	13 (46)	10 (37)	
	Yes, both	1 (4)	1 (4)	
**Comorbidity, n (%)**				
	DM1^g^	1 (4)	1 (4)	
	DM2^h^	6 (21)	2 (7)	
	Hypertension	10 (36)	4 (15)	
	CV disease^i^	4 (14)	7 (26)	
	TE disease^j^	6 (21)	6 (22)	
	Depression/low mood	2 (7)	3 (11)	
**Regular use/abuse, n (%)**				
	None	9 (32)	9 (33)	
	Tobacco	14 (50)	15 (56)	
	Alcohol	13 (46)	11 (41)	
	Illegal drugs	0 (0)	1 (4)	

^a^SCI: spinal cord injury.

^b^TSCI: traumatic spinal cord injury.

^c^NTSCI: nontraumatic spinal cord injury.

^d^Level of injury: location of the injury in the spinal cord (C: cervical, T: thoracic, and S: sacrum).

^e^AIS grade: completeness/severity of the injury.

^f^PI: pressure injury.

^g^DM1: diabetes mellitus type 1.

^h^DM2: diabetes mellitus type 2.

^i^CV disease: cardiovascular disease.

^j^TE disease: thromboembolic disease.

### Pressure Injuries at Baseline

In the VCG, 32% (9/28) of the PIs were category 2, 50% (14/28) category 3, 11% (3/28) category 4, and 7% (2/28) could not be categorized at the time of inclusion. The distribution in the RCG was 52% (14/27) were category 2, 22% (6/27) category 3, 19% (5/27) category 4, and 7% (2/27) were unstageable.

Most of the PIs were located at the ischial tuberosities: 50% (14/28) in the VCG and 33% (9/27) in the RCG. At the sacrum-gluteal cleft, PIs occurred in 32% (9/28) of the participants in the VCG and 48% (13/27) in the RCG.

### Health-Related Quality of Life

The SF-36 scale, the UK version of the EQ-5D scale, and the ISCI-QoL-BDS basic data set were used to measure and compare changes in HRQoL. Performing a linear regression analysis, comparing the 2 groups with adjustment for baseline, did not yield any significant differences regarding HRQoL, as shown in Multimedia Appendix 1 (imputed data). Multimedia Appendix 2 shows the complete data.

### Healing

**Figure 4:**
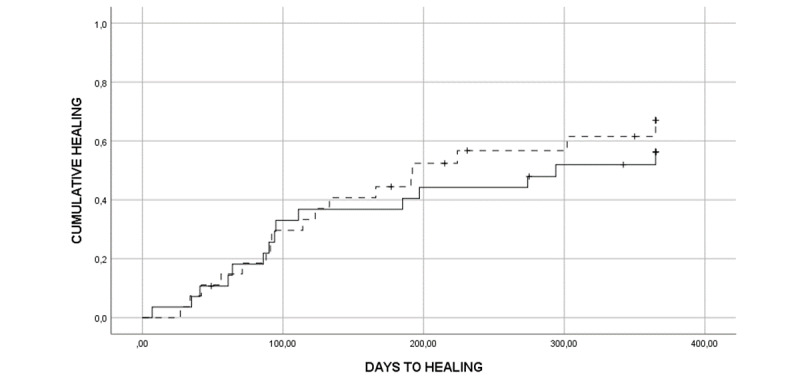
Kaplan-Meier plot showing time to healing in the two groups (videoconference: solid line; regular care group: dotted line).

A total of 67% (37/55) of all PIs healed completely during follow-up: 64% (18/28) in the VCG versus 70% (19/27) in the RCG. Mean reduction in ulcer volume in the VCG was 79% versus 85% in the RCG. No significant difference in the 2 groups were found (P=.32). The median time to healing in the VCG was 275 days (95% CI 111.18-438.83) versus 192 days (95% CI 113.71-270.29) in the RCG. A Kaplan-Meier plot (Figure 4) with a logrank test regarding time to healing did not show any significant difference between the 2 groups (P=.56). Figure 4 displays time to healing in both groups.

### Interaction, Satisfaction, and Safety

A total of 85% (47/55) of the included participants responded to the feedback form, 86% (24/28) in the VCG and 85% (23/27) in the RCG. No significant differences were found in interaction, satisfaction or safety, and the estimated mean differences were minor. Table 2 shows the mean difference between the RCG and VCG, with corresponding confidence intervals and P values. The district nurses were also asked to report their experienced interaction, satisfaction, and safety with the follow-up. A total of 45% (24/55) of the nurses responded, 52% (14/28) in the VCG and 38% (10/27) in the RCG. No significant differences were found in the 2 groups.

**Table 2:** Comparison of interaction, satisfaction, and safety experienced by participants and district nurses as reported at follow-up.

		Mean difference^a^	95% CI	P value^b^
**Participants**				
	Planning	–0.08	–0.78 to 0.62	0.82
	Implementation	–0.04	–0.73 to 0.81	0.91
	Interaction	0.14	–0.59 to 0.87	0.70
	Participation	–0.13	–0.67 to 0.94	0.74
	Safety	–0.01	–0.77 to 0.76	0.99
	Usefulness	–0.19	0.97 to 0.60	0.63
	Overall satisfaction	0.11	0.66 to 0.88	0.78
**District nurses**				
	Planning	0.21	–0.41 to 0.82	0.49
	Implementation	0.04	–0.55 to 0.63	0.88
	Interaction	0.33	–0.32 to 0.99	0.30
	Participation	0.00	–0.60 to 0.60	1.00
	Safety	0.16	0.41 to 0.74	0.56
	Usefulness	–0.15	0.87 to 0.56	0.65
	Overall satisfaction	–0.16	–0.68 to 0.36	0.52

^a^Mean difference: difference in mean values (regular care group minus videoconference group).

^b^Based on an independent *t* test.

## Discussion

### Principal Findings

SCI has a huge impact on the individual, the family, and the health care system. Regular contact with specialized health care is required for the condition itself as well as the frequent related complications such as PI. Thus, there is an urgent need to secure availability of high-quality services for patients who live far from specialists and require long-term follow-up [5,34]. Individuals with SCI and PI require frequent outpatient care to monitor their wounds [34]. Long travel distances to receive treatment, resulting in time-consuming transport, can attribute to greater morbidity [6]. To our knowledge, this is the first randomized controlled study using videoconferencing to provide long-term treatment to persons with PI. The results from our study indicate that regular home-based videoconferences are as safe for patients and their district nurses as conventional care with in-person attendance.

According to our study, the HRQoL was not dependent of the type of health service offered. We still find it relevant to mention that the estimated mean difference was in favor of the VCG for 12 out of 13 HRQoL scores. There were no substantial differences between the analyses based on the imputed data (Multimedia Appendix 1) and the complete case analysis (Multimedia Appendix 2).

In this study, the 2 groups were evenly distributed by gender, age, PI occurrence, and PI location. There were no significant differences regarding healing between the 2 groups. Looking at the Kaplan-Meier plot (Figure 4), the 2 curves follow each other very closely, at least for the first 200 days, indicating that the videoconference service was as efficient as the conventional follow-up. All participants and their district nurses were given similar guidance regarding nutrition, skin care, PI prevention, position change, and pressure relieving mattresses and cushions, and an individual treatment plan was established for each participant in both groups [27].

We also investigated the association between potential risk factors and time to healing as a post hoc analysis. Interestingly, overall comorbidities did not show any association regarding time to heal. Due to low number of concomitant diseases among the participants in our study (Table 1), further substudies could not be performed.

Participants in both groups and their district nurses reported acceptable levels of experienced interaction and satisfaction, with no significant differences regarding the follow-up. This indicates videoconference consultations offer satisfactory remote interaction with the district nurses as compared to regular follow-up. However, we believe a larger study with a noninferiority design would be warranted to establish this.

There is a lack of studies regarding PI and long-term follow-up in the literature. Based on the number of nonhealing PIs in our study, a longer follow-up period may be an interesting topic for future research. We also think that the issue of nonhealing PI should be further explored, no matter the mode of follow-up intervention.

Telemedicine has been widely adapted in many fields of medicine, especially in recent years. We believe that this should also be the case for rehabilitation and that individualized follow-up where a hybrid solution of video communication and conventional consultations is used, may be a promising path for the future.

### Limitations

When the present study was designed, we based our sample size calculation on an investigation of HRQoL. However, we do not have sufficient statistical power to provide conclusive evidence regarding the rest of the comparisons we performed in this study.

### Comparison With Prior Work

This study is the first randomized, controlled, multidisciplinary long-term study using videoconference as mode of administration of treatment to provide care to persons with SCI and PI [15]. Videoconference consultations seem to be an acceptable solution concerning treatment and follow-up. Our study shows feasibility and efficacy in the examined population. However, the heterogeneity regarding participants, modalities, and the level of mixed evidence in previous research makes it difficult to compare with prior work [15,35]. This is also in line with previous research [13,15,17,20,21,36].

### Conclusion

Videoconference in a patient’s home ensures safe and efficient quality of care without any reduction in HRQoL, PI healing, or satisfaction as compared to conventional outpatient care at the hospital. Long-term videoconference at home under these circumstances ensures interaction with patients and district nurses and assures they receive relevant information on-site. Further research should assess and compare the value of videoconference for routine long-term care, such as managing spasticity, urinary tract and bowel needs, and chronic pain.

### Data Archiving

The dataset is stored in a locked and fireproof research cabinet at the research department, Sunnaas Rehabilitation Hospital, Norway, and can be made available on request according to the Norwegian Data and Telecommunications Authority’s requirements for safe information flow [26].

## Appendix

Multimedia Appendix 1 Differences in health-related quality of life between the two groups from baseline to end of follow-up, based on imputed data.

Multimedia Appendix 2 Differences in health-related quality of life between the two groups from baseline to end of follow-up, based on actual data.

Multimedia Appendix 3 CONSORT-eHEALTH checklist (V 1.6.2).

## Abbreviations

AIS: American Spinal Injury Association (ASIA) Impairment Scale
ASIA: American Spinal Injury Association
CONSORT: Consolidated Standards of Reporting Trials
EQ-5D: Five Dimensions European Quality of Life scale
HRQoL: health-related quality of life
ISCI-QoL-BDS: International Spinal Cord Injury Quality of Life Basic Data Set
PI: pressure injury
RCG: regular care group
SCI: spinal cord injury
SF-36: 36-item Short Form Health Survey
SPIRIT: Standard Protocol Items: Recommendations for Interventional Trials
TIDieR: Template for Intervention Description and Replication
VCG: videoconference group
