# Supplementary material for: Videoconferencing in Pressure Injury: Randomized Controlled Telemedicine Trial in Patients With Spinal Cord Injury
Source: JMIR Form Res. 2022 Apr 19;6(4):e27692. doi: 10.2196/27692 (PMC9066320; doi:10.2196/27692)
Supplement: Multimedia Appendix 1 [file formative_v6i4e27692_app1.docx]

Multimedia Appendix 1. Differences in health-related quality of life between the two groups from baseline to end of follow-up, based on imputed data.

| **QUESTIONNAIRE** | | **GROUP** | **BASELINE** | | **END OF FOLLOW-UP** | | **Estimated mean difference** | **95% CI** | ***P* value** |
| --- | --- | --- | --- | --- | --- | --- | --- | --- | --- |
|  |  |  | **Mean** | **95% CI** | **Mean** | **95% CI** |  |  |  |
| **ISCI-QoL-**  **BDS** | Overall health | VCG | 6.15 | 5.26 to 7.04 | 6.26 | 5.41 to 7.11 | -0.93 | -2.13 to 0.28 | 0.13 |
|  |  | RCG | 5.60 | 4.52 to 6.69 | 5.07 | 3.93 to 6.21 |  |  |  |
|  | Physical health | VCG | 5.22 | 4.28 to 6.16 | 6.07 | 5.16 to 6.99 | -0.99 | -2.29 to 0.32 | 0.14 |
|  |  | RCG | 5.05 | 4.10 to 6.00 | 5.03 | 3.90 to 6.15 |  |  |  |
|  | Mental health | VCG | 7.37 | 6.65 to 8.09 | 7.59 | 6.73 to 8.45 | -1.09 | -2.56 to 0.38 | 0.15 |
|  |  | RCG | 5.95 | 4.78 to 7.13 | 5.79 | 4.38 to 7.20 |  |  |  |
| **SF-36** | Physical functioning | VCG | 38.97 | 29.86 to 48.07 | 34.33 | 24.08 to 44.59 | -5.37 | -18.44 to 7.69 | 0.42 |
|  |  | RCG | 32.04 | 22.71 to 41.37 | 26.51 | 16.67 to 36.35 |  |  |  |
|  | Physical role | VCG | 24.97 | 10.67 to 39.27 | 37.45 | 20.37 to 54.54 | 1.60 | -22.66 to 25.87 | 0.90 |
|  |  | RCG | 36.25 | 21.26 to 51.24 | 41.39 | 22.38 to 60.41 |  |  |  |
|  | Pain | VCG | 51.32 | 37.41 to 65.23 | 63.00 | 49.57 to 76.42 | -6.45 | -20.56 to 7.67 | 0.37 |
|  |  | RCG | 48.45 | 37.66 to 59.25 | 54.88 | 43.40 to 66.37 |  |  |  |
|  | General health perceptions | VCG | 57.95 | 49.62 to 66.62 | 61.80 | 54.59 to 69.00 | -6.92 | -16.84 to 3.00 | 0.17 |
|  |  | RCG | 50.52 | 41.20 to 59.84 | 52.24 | 43.62 to 60.86 |  |  |  |
|  | Vitality | VCG | 51.11 | 44.30 to 57.93 | 58.18 | 48.90 to 67.46 | -5.72 | -17.49 to 6.06 | 0.34 |
|  |  | RCG | 47.95 | 41.23 to 54.67 | 51.74 | 43.25 to 60.23 |  |  |  |
|  | Social functioning | VCG | 57.48 | 46.57 to 68.39 | 68.54 | 55.28 to 81.80 | -9.90 | -29.90 to 10.10 | 0.33 |
|  |  | RCG | 70.19 | 58.94 to 81.45 | 63.34 | 46.58 to 80.11 |  |  |  |
|  | Emotional problems | VCG | 52.90 | 36.34 to 69.46 | 70.56 | 53.35 to 87.76 | -16.77 | -39.73 to 6.19 | 0.15 |
|  |  | RCG | 65.38 | 48.57 to 82.20 | 57.56 | 39.78 to 75.34 |  |  |  |
|  | Mental health | VCG | 70.73 | 64.57 to 76.88 | 76.64 | 67.36 to 85.93 | -4.03 | -14.80 to 6.75 | 0.46 |
|  |  | RCG | 70.35 | 63.11to 77.58 | 72.41 | 64.26 to 80.56 |  |  |  |
| **EQ-5D** | EQ-5 dimensions | VCG | 0.09 | 0.01 to 0.16 | 0.06 | -0.02 to 0.13 | -0.05 | -0.16 to 0.06 | 0.39 |
|  |  | RCG | 0.09 | 0.03 to 0.15 | 0.04 | -0.06 to 0.13 |  |  |  |
|  | EQ-VAS | VCG | 57.19 | 47.73 to 66.64 | 62.34 | 54.47 to 70.21 | -4.89 | -15.24 to 5.46 | 0.35 |
|  |  | RCG | 57.19 | 48.54 to 65.85 | 57.45 | 49.21 to 65.69 |  |  |  |

^VCG= Videoconference Group, RCG= Regular care group, CI = confidence interval. SCI QoL BDS= Spinal Cord Injury-Quality of Life Basic Data Set. EQ-5D= EuroQuality of life-5 Dimensions.^

^SF-36= The Short Form (36) Health Survey.^
