# Supplementary material for: Videoconferencing in Pressure Injury: Randomized Controlled Telemedicine Trial in Patients With Spinal Cord Injury
Source: JMIR Form Res. 2022 Apr 19;6(4):e27692. doi: 10.2196/27692 (PMC9066320; doi:10.2196/27692)
Supplement: Multimedia Appendix 2 [file formative_v6i4e27692_app2.docx]

Multimedia Appendix 2. Differences in health-related quality of life between the two groups from baseline to end of follow-up, based on actual data.

| **QUESTIONNAIRE** | | **GROUP** | **BASELINE** | | | **END OF FOLLOW-UP** | | | **Estimated mean difference** | **95% CI** | ***P* value** |
| --- | --- | --- | --- | --- | --- | --- | --- | --- | --- | --- | --- |
|  |  |  | **n** | **Mean** | **95% CI** | **n** | **Mean** | **95% CI** |  |  |  |
| **ISCI-QoL** | Overall health | VCG | 27 | 6.15 | 5.26 to 7.04 | 27 | 6.26 | 5.41 to 7.11 | 0.494 | 0.251 to 0.762 | 0.000 |
|  |  | RCG | 23 | 5.87 | 4.75 to 6.99 | 23 | 5.30 | 4.12 to 6.49 |  |  |  |
|  | Physical health | VCG | 27 | 5.22 | 4.28 to 6.16 | 27 | 6.07 | 5.16 to 6.99 | 0.328 | 0.067 to 0.664 | 0.017 |
|  |  | RCG | 24 | 5.21 | 4.27 to 6.15 | 24 | 5.04 | 3.88 to 6.20 |  |  |  |
|  | Mental health | VCG | 27 | 7.37 | 6.65 to 8.09 | 27 | 7.59 | 6.73 to 8.45 | 0.434 | 0.221 to 0.864 | 0.001 |
|  |  | RCG | 24 | 6.17 | 5.01 to 7.32 | 24 | 5.83 | 4.39 to 7.28 |  |  |  |
| **SF-36** | Physical functioning | VCG | 23 | 40.87 | 31.36 to 50.38 | 23 | 35.43 | 25.13 to 45.74 | 0.386 | 0.105 to 4.51 | 0.009 |
|  |  | RCG | 21 | 34.35 | 23.70 to 44.99 | 21 | 24.29 | 14.44 to 34.14 |  |  |  |
|  | Physical role | VCG | 23 | 24.64 | 9.56 to 39.71 | 23 | 37.32 | 19.54 to 55.10 | 0.196 | -0.196 to 0.589 | 0.218 |
|  |  | RCG | 21 | 40.48 | 23.04 to 57.91 | 21 | 42.86 | 22.45 to 63.26 |  |  |  |
|  | Pain | VCG | 23 | 53.48 | 38.94 to 68.02 | 23 | 65.22 | 51.07 to 79.37 | 0.643 | 0.404 to 0.862 | 0.000 |
|  |  | RCG | 22 | 52.27 | 41.12 to 63.43 | 22 | 55.23 | 44.45 to 66.01 |  |  |  |
|  | General health perceptions | VCG | 23 | 57.39 | 49.36 to 65.43 | 23 | 61.09 | 53.54 to 68.63 | 0.471 | 0.206 to 0.760 | 0.001 |
|  |  | RCG | 20 | 56.25 | 47.47 to 65.03 | 20 | 51.75 | 42.54 to 60.96 |  |  |  |
|  | Vitality | VCG | 22 | 50.45 | 42.90 to 58.01 | 22 | 58.18 | 47.88 to 68.48 | 0.193 | -0.151 to 0.662 | 0.212 |
|  |  | RCG | 22 | 50.98 | 44.40 to 57.57 | 22 | 53.26 | 45.06 to 61.46 |  |  |  |
|  | Social functioning | VCG | 23 | 57.61 | 46.11 to 69.10 | 23 | 72.28 | 60.31 to 84.25 | 0.350 | 0.049 to 0.831 | 0.028 |
|  |  | RCG | 22 | 75.00 | 65.48 to 84.52 | 22 | 65.91 | 49.76 to 82.06 |  |  |  |
|  | Emotional problems | VCG | 23 | 52.17 | 33.82 to 70.52 | 23 | 68.12 | 49.43 to 86.80 | 0.330 | 0.026 to 0.637 | 0.034 |
|  |  | RCG | 21 | 71.43 | 53.29 to 89.56 | 21 | 57.14 | 38.47 to 75.81 |  |  |  |
|  | Mental health | VCG | 22 | 71.45 | 65.30 to 77.61 | 22 | 76.36 | 66.56 to 86.17 | 0.440 | 0.203 to 0.978 | 0.004 |
|  |  | RCG | 21 | 74.86 | 68.31 to 81.40 | 21 | 73.71 | 66.82 to 80.61 |  |  |  |
| **EQ-5D** | EQ-5 dimensions | VCG | 26 | 0.0885 | 0.0103 to 0.167 | 26 | 0.0897 | 0.0266 to 0.153 | 0.199 | -0.097 to 0.527 | 0.172 |
|  |  | RCG | 23 | 0.0793 | 0.00516 to 0.153 | 23 | 0.0467 | -0.0548 to 0.148 |  |  |  |
|  | EQ-VAS | VCG | 26 | 57.27 | 47.45 to 67.090 | 26 | 61.81 | 53.85 to 69.77 | 0.354 | 0.077 to 0.572 | 0.011 |
|  |  | RCG | 25 | 59.28 | 51.44 to 67.12 | 25 | 57.56 | 49.22 to 65.90 |  |  |  |

^n= number of answers. CI = confidence interval. The mean difference is the difference from baseline to end of follow-up. SCI QoL= Spinal Cord Injury-Quality of Life basic form. EQ-5D= EuroQuality of life-5 Dimensions. SF-36= The Short Form (36) Health Survey.^
