# Supplementary material for: Videoconferencing in Pressure Injury: Randomized Controlled Telemedicine Trial in Patients With Spinal Cord Injury
Source: JMIR Form Res. 2022 Apr 19;6(4):e27692. doi: 10.2196/27692 (PMC9066320; doi:10.2196/27692)
Supplement: Multimedia Appendix 3 [file formative_v6i4e27692_app3.pdf]

CONSORT-EHEALTH Checklist V1.6.2 Report	Manuscript Number	27692
**(based on CONSORT-EHEALTH V1.6), available at [http://tinyurl.com/consort-ehealth-v1-6].**		
**Date completed**		
2/18/2022 20:39:04		
**by**		
Ingebjørg Irgens		
Videoconferencing in Pressure Injury: Randomized Controlled Telemedicine Trial in Patients With Spinal Cord Injury		
TITLE		
**1a-i) Identify the mode of delivery in the title**		
The manuscript title contains the used technical solution (videoconference), and the overall phrase used to describe such solutions (telemedicine)		
**1a-ii) Non-web-based components or important co-interventions in title**		
The article address videoconference		
**1a-iii) Primary condition or target group in the title**		
Pressure Injury in Patients With Spinal Cord Injury		
ABSTRACT		
**1b-i) Key features/functionalities/components of the intervention and comparator in the METHODS section of the ABSTRACT**		
A total of... were randomized to a videoconference group or a regular care group.		
**1b-ii) Level of human involvement in the METHODS section of the ABSTRACT**		
The number of participants, and the health care professionals are reported		
**1b-iii) Open vs. closed, web-based (self-assessment) vs. face-to-face assessments in the METHODS section of the ABSTRACT**		
Addressed in the objectives and in the methods part		
**1b-iv) RESULTS section in abstract must contain use data**		
Number of participants are reported in the methods part of the abstract. In the result part, the main and secondary outcomes are reported		
**1b-v) CONCLUSIONS/DISCUSSION in abstract for negative trials**		
The findings are addressed in the conclusion		
INTRODUCTION		
**2a-i) Problem and the type of system/solution**		
The article is addressing the potential of videoconferencing at the patient`s home regarding health care accessibility for persons in need of long-term follow-up		
**2a-ii) Scientific background, rationale: What is known about the (type of) system**		
The knowledge are mixed, therefore new studies are needed		
**Does your paper address CONSORT subitem 2b?**		
the hypothesis is that videoconference increases health related quality of life in persons with spinal cord injury and wounds, and that satisfaction and interaction would benefit from the use of this technology		
METHODS		
**3a) CONSORT: Description of trial design (such as parallel, factorial) including allocation ratio**		
The study is a randomized controlled trial whit a two-group allocation		
**3b) CONSORT: Important changes to methods after trial commencement (such as eligibility criteria), with reasons**		
No changes in methodology have been performed since the study was initiated		
**3b-i) Bug fixes, Downtimes, Content Changes**		
Upgrading of the software have been performed according to standard procedures		
**4a) CONSORT: Eligibility criteria for participants**		
The eligibility criteria were having a spinal cord injury and an ongoing pressure injury, as well as being 18 years or older, and living in Norway.		
**4a-i) Computer / Internet literacy**		
Computer/ internet literacy is not relevant in this article		
**4a-ii) Open vs. closed, web-based vs. face-to-face assessments:**		
Recruitment via answers on a questionnaire, and from the spinal cord units. Face-to-face consultations in both groups		
**4a-iii) Information giving during recruitment**		
Oral and written information were given to all potential participants.		
**4b) CONSORT: Settings and locations where the data were collected**		
The data collection were performed at the spinal cord units by the wound physicians and the wound care nurses, and in the municipalities, performed by the district nurses, and transferred to the main researcher.		
**4b-i) Report if outcomes were (self-)assessed through online questionnaires**		
Outcomes were a combination of assessments by the research team, the district nurses, and self-assessment by the participants. The self-assessments were performed via questionnaires and forms, sent via post.		
**4b-ii) Report how institutional affiliations are displayed**		
The affiliations were two Norwegian spinal cord units.		
**5) CONSORT: Describe the interventions for each group with sufficient details to allow replication, including how and when they were actually administered**		
**5-i) Mention names, credential, affiliations of the developers, sponsors, and owners**		
This was not a topic in the protocol. Further information regarding the research project can be found in the protocol article, Doi:10.1186/s13063-019-3185-y.		
**5-ii) Describe the history/development process**		
A feasibility study was performed in advance of the research project, article can be found at doi:10.4045/tidsskr.15.0770.		
**5-iii) Revisions and updating**		
The project was evaluated by an external monitoring group. The intervention was frozen during the trial.		
**5-iv) Quality assurance methods**		
In the feasibility study, we had external researcher following the project to secure accuracy and quality of the project. This research is presented in the book Velferdsteknologi, Cappelen Damm Akademisk (2019), Chapter 3. (Editor Ingunn Moser).		
**5-v) Ensure replicability by publishing the source code, and/or providing screenshots/screen-capture video, and/or providing flowcharts of the algorithms used**		
No source code or algorithms used.		
**5-vi) Digital preservation**		
Archived according to the regional ethical committee and the legislation of research projects in Norway		
**5-vii) Access**		
The software is free to use.		
**5-viii) Mode of delivery, features/functionalities/components of the intervention and comparator, and the theoretical framework**		
This can be found in the protocol article, Doi:10.1186/s13063-019-3185-y.		
**5-ix) Describe use parameters**		
The videoconference group, twice a month. The regular care group according to request from the participants or their district nurses.		
**5-x) Clarify the level of human involvement**		
The multidisciplinary team at the outpatient clinic guided the local care providers (district nurses) in both groups.		
**5-xi) Report any prompts/reminders used**		
Each participant in the videoconference group got their personal log-on number, and when the wound care nurse called this number, the participants had to approve the consultation before access for the multidisciplinary wound team.		
**5-xii) Describe any co-interventions (incl. training/support)**		
The district nurses in both groups were guided in prevention and treatment.		
**6a) CONSORT: Completely defined pre-specified primary and secondary outcome measures, including how and when they were assessed**		
Health related quality of life and volume of the pressure injury were assessed at baseline and at end of follow-up/ when the pressure injury had healed.		
**6a-i) Online questionnaires: describe if they were validated for online use and apply CHERRIES items to describe how the questionnaires were designed/deployed**		
No online questionnaires. However, all quality of life questionnaires used were validated.		
**6a-ii) Describe whether and how "use" (including intensity of use/dosage) was defined/measured/monitored**		
Not necessary in this study		
**6a-iii) Describe whether, how, and when qualitative feedback from participants was obtained**		

Ongoing during the trial, and qualitative in-depth interviews were performed at end of study.		
**6b) CONSORT: Any changes to trial outcomes after the trial commenced, with reasons**		
The data collection were performed at the spinal cord units by the wound physicians and the wound care nurses, and in the municipalities, performed by the district nurses, and transferred to the main researcher.		
**7a) CONSORT: How sample size was determined**		
**7a-i) Describe whether and how expected attrition was taken into account when calculating the sample size**		
Sample size were calculated on investigation of health related quality of life.		
**7b) CONSORT: When applicable, explanation of any interim analyses and stopping guidelines**		
Health related quality of life and volume of the pressure injury were assessed at baseline and at end of follow-up/ when the pressure injury had healed.		
**8a) CONSORT: Method used to generate the random allocation sequence**		
The random number generator in the statistical software SPSS, version 25 was used.		
**8b) CONSORT: Type of randomisation; details of any restriction (such as blocking and block size)**		
Blocking and stratification.		
**9) CONSORT: Mechanism used to implement the random allocation sequence (such as sequentially numbered containers), describing any steps taken to conceal the sequence until interventions were assigned**		
The random allocation sequence is described in the protocol article, Doi:10.1186/s13063-019-3185-y.		
**10) CONSORT: Who generated the random allocation sequence, who enrolled participants, and who assigned participants to interventions**		
An external statistician.		
**11a) CONSORT: Blinding - If done, who was blinded after assignment to interventions (for example, participants, care providers, those assessing outcomes) and how**		
**11a-i) Specify who was blinded, and who wasn't**		
Non-blinded, due to the design of the study, however, only the random ID number were known during the analyses.		
**11a-ii) Discuss e.g., whether participants knew which intervention was the "intervention of interest" and which one was the "comparator"**		
The participants knew, due to the design of the study.		
**11b) CONSORT: If relevant, description of the similarity of interventions**		
Not relevant in this study.		
**12a) CONSORT: Statistical methods used to compare groups for primary and secondary outcomes**		
This is described in the protocol article, Doi:10.1186/s13063-019-3185-y.		
**12a-i) Imputation techniques to deal with attrition / missing values**		
missing data were handled by multiple imputation.		
**12b) CONSORT: Methods for additional analyses, such as subgroup analyses and adjusted analyses**		
No subgroup analyses were performed.		
RESULTS		
**13a) CONSORT: For each group, the numbers of participants who were randomly assigned, received intended treatment, and were analysed for the primary outcome**		
57 participants were included, 28 in each group.		
**13b) CONSORT: For each group, losses and exclusions after randomisation, together with reasons**		
3 participants died, one after inclusion, but before start. Further one participant in each of the two groups died during the follow-up, all deaths due to acute cardiovascular illness (2)/ pneumonia (1).		
**13b-i) Attrition diagram**		
All participants used the technical solutions during the study period.		
**14a) CONSORT: Dates defining the periods of recruitment and follow-up**		
Start March 6th 2016, end October 19th 2019.		
**14a-i) Indicate if critical "secular events" fell into the study period**		
No critical events happened. Three participants died ,due to diseases not related to the intervention illness.		
**14b) CONSORT: Why the trial ended or was stopped (early)**		
The trial ended when the last participant were finished with the follow-up/ the pressure injury was healed.		
**15) CONSORT: A table showing baseline demographic and clinical characteristics for each group**		
This is described in the paper.		
**15-i) Report demographics associated with digital divide issues**		
This is described in the paper.		
**16a) CONSORT: For each group, number of participants (denominator) included in each analysis and whether the analysis was by original assigned groups**		
**16-i) Report multiple "denominators" and provide definitions**		
This is described in the paper.		
**16-ii) Primary analysis should be intent-to-treat**		
This is described in the paper.		
**17a) CONSORT: For each primary and secondary outcome, results for each group, and the estimated effect size and its precision (such as 95% confidence interval)**		
This is described in the paper.		
**17a-i) Presentation of process outcomes such as metrics of use and intensity of use**		
Not important in this paper. Will be described in an upcoming paper.		
**17b) CONSORT: For binary outcomes, presentation of both absolute and relative effect sizes is recommended**		
Not relevant in this paper.		
**18) CONSORT: Results of any other analyses performed, including subgroup analyses and adjusted analyses, distinguishing pre-specified from exploratory**		
This is described in the paper.		
**18-i) Subgroup analysis of comparing only users**		
This is of no interest in the present paper.		
**19) CONSORT: All important harms or unintended effects in each group**		
This is described in the paper.		
**19-i) Include privacy breaches, technical problems**		
Will be described in an upcoming paper.		
**19-ii) Include qualitative feedback from participants or observations from staff/researchers**		
In-depth interviews are performed, and will be presented in an upcoming paper.		
DISCUSSION		
**20) CONSORT: Trial limitations, addressing sources of potential bias, imprecision, multiplicity of analyses**		
**20-i) Typical limitations in ehealth trials**		
This is described in the paper.		
**21) CONSORT: Generalisability (external validity, applicability) of the trial findings**		
**21-i) Generalizability to other populations**		
This is described in the paper.		
**21-ii) Discuss if there were elements in the RCT that would be different in a routine application setting**		
This will be discussed in an upcoming paper.		
**22) CONSORT: Interpretation consistent with results, balancing benefits and harms, and considering other relevant evidence**		
**22-i) Restate study questions and summarize the answers suggested by the data, starting with primary outcomes and process outcomes (use)**		
This is described in the paper.		
**22-ii) Highlight unanswered new questions, suggest future research**		
This is described in the paper.		
Other information		
**23) CONSORT: Registration number and name of trial registry**		
ClinicalTrials.gov NCT02800915 and CRISTIN 545284.		
**24) CONSORT: Where the full trial protocol can be accessed, if available**		
The protocol article, Doi:10.1186/s13063-019-3185-y.		
**25) CONSORT: Sources of funding and other support (such as supply of drugs), role of funders**		
Not relevant in this study.		
**X26-i) Comment on ethics committee approval**		

the study was approved by the Regional ethical committee before start.		
**x26-ii) Outline informed consent procedures**		
Consent obtained in person.		
**X26-iii) Safety and security procedures**		
The study followed the Norwegian privacy legislation/GDPR legislation.		
**X27-i) State the relation of the study team towards the system being evaluated**		
The main researcher was granted by the DAM Foundation.		
